# Risk factors for recognized and unrecognized SARS-CoV-2 infection: a seroepidemiologic analysis of the Prospective Urban Rural Epidemiology (PURE) study

**DOI:** 10.1128/spectrum.01492-23

**Published:** 2024-01-12

**Authors:** Darryl P. Leong, Mark Loeb, Prem K. Mony, Sumathy Rangarajan, Maha Mushtaha, Matthew S. Miller, Mary Dias, Sergey Yegorov, Mamatha V, Ozge Telci Caklili, Ahmet Temizhan, Andrzej Szuba, Marc Evans M. Abat, Nafiza Mat-Nasir, Maria Luz Diaz, Hamda Khansaheb, Patricio Lopez-Jaramillo, MyLinh Duong, Koon K. Teo, Paul Poirier, Gustavo Oliveira, Álvaro Avezum, Salim Yusuf

**Affiliations:** 1 The Population Health Research Institute, McMaster University, Hamilton, Canada; 2 Department of Medicine, McMaster University, Hamilton, Canada; 3 Department of Pathology and Molecular Medicine, McMaster University, Hamilton, Canada; 4 Department of Health Research Methods, Evidence, and Impact, McMaster University, Hamilton, Canada; 5 Division of Epidemiology and Population Health, St. John’s Research Institute, St. John’s Medical College, Bangalore, India; 6 Department of Biochemistry and Biomedical Sciences, McMaster University, Hamilton, Canada; 7 Department of Microbiology and Infectious Diseases, St. John’s Medical College, Bangalore, India; 8 Department of Endocrinology and Metabolism, Istanbul University, Istanbul, Turkey; 9 Cardiology Department, Ankara Bilkent City Hospital, Ankara, Turkey; 10 Department of Angiology, Hypertension and Diabetology, Wroclaw Medical University, Wroclaw, Poland; 11 Department of Medicine, Philippine General Hospital, Manila, Philippines; 12 Department of Primary Care Medicine, Universiti Teknologi MARA (UiTM), Petaling Jaya, Malaysia; 13 Estudios Clinicos Latinamérica (ECLA), Instituto Cardiovascular de Rosario, Rosario, Argentina; 14 Dubai Academic Health Corporation, Umm Hurair, Dubai, UAE; 15 MASIRA Research Institute, Medical School, Universidad de Santander, Bucaramanga, Colombia; 16 Faculté de pharmacie, Université Laval, Québec, Canada; 17 Hospital Alemão Oswaldo Cruz, São Paolo, Brazil; National Institute of Allergy and Infectious Diseases, Baltimore, Maryland, USA

**Keywords:** SARS-CoV-2, seroepidemiology, COVID-19, low-income countries, pandemic, transmission

## Abstract

There are limited data on individual risk factors for SARS-CoV-2 infection (including unrecognized infection). In this seroepidemiologic substudy of an ongoing prospective cohort study of community-dwelling adults, participants were thoroughly characterized pre-pandemic. The SARS-CoV-2 infection was ascertained by serology. Among 8,719 participants from 11 high-, middle-, and low-income countries, 3,009 (35%) were seropositive for SARS-CoV-2. Characteristics independently associated with seropositivity were younger age (odds ratio, OR; 95% confidence interval, CI, per five-year increase: 0.95; 0.91–0.98) and body mass index >25 kg/m^2^ (OR, 95% CI: 1.16, 1.01–1.34). Smoking (as compared with never smoking, OR, 95% CI: 0.83, 0.70–0.97) and COVID-19 vaccination (OR, 95% CI: 0.70, 0.60–0.82) were associated with a reduced risk of seropositivity. Among seropositive participants, 83% were unaware of having been infected with SARS-CoV-2. Seropositivity and a lack of awareness of infection were more common in lower-income countries. The COVID-19 vaccination reduces the risk of SARS-CoV-2 infection (including recognized and unrecognized infections). Overweight or obesity is an independent risk factor for SARS-CoV-2 infection. Infection and lack of infection awareness are more common in lower-income countries.

**IMPORTANCE:**

In this large, international study, evidence of SARS-CoV-2 infection was obtained by testing blood specimens from 8,719 community-dwelling adults from 11 countries. The key findings are that (i) the large majority (83%) of community-dwelling adults from several high-, middle-, and low-income countries with blood test evidence of SARS-CoV-2 infection were unaware of this infection—especially in lower-income countries; and (ii) overweight/obesity predisposes to SARS-CoV-2 infection, while COVID-19 vaccination is associated with a reduced risk of SARS-CoV-2 infection. These observations are not attributable to other individual characteristics, highlighting the importance of the COVID-19 vaccination to prevent not only severe infection but possibly any infection. Further research is needed to understand the mechanisms by which overweight/obesity might increase the risk of SARS-CoV-2 infection.

## INTRODUCTION

Severe acute respiratory syndrome coronavirus-2 (SARS-CoV-2) has had an unprecedented impact on populations globally. The virus has remained an important cause of morbidity and mortality since the COVID-19 pandemic was declared.

Although there are many studies reporting factors associated with severe COVID-19 infection (e.g., fatal or requiring mechanical ventilation or hospitalization), there are few data on individual-level factors associated with susceptibility to any SARS-CoV-2 infection (including asymptomatic or subclinical infection). Meyerowitz et al. screened databases to identify research on host factors affecting SARS-CoV-2 infection (1). They identified older age and immunocompromise as risk factors. A systematic review of household SARS-CoV-2 transmission found that secondary infection rates were higher among contacts with co-morbidities than among contacts without co-morbidities (2). However, most cohort studies did not collect comprehensive data on patients’ or contacts’ characteristics (3). Therefore, there is little information on individual-level factors associated with increased susceptibility to SARS-CoV-2 infection. Also, existing evidence on SARS-CoV-2 risk factors is mostly from high-income countries, with no multi-national studies on risk factors for SARS-CoV-2 infection. This evidence gap is important because the greatest burden from COVID-19 is in middle- and low-income countries (4), which represent most of the world’s population.

Ascertainment of SARS-CoV-2 infection by patient self-report, self-referral for COVID-19 testing, or a need for hospitalization is unreliable because of the high rate of asymptomatic or unrecognized infection, limited reporting of home test results, and variability in the availability of tests and hospital resources. Systematically conducted serology is the only way to comprehensively identify individuals with a past SARS-CoV-2 infection. The primary aim of this study was to identify individual-level characteristics that are associated with a higher risk for serologic evidence of SARS-CoV-2 infection—both symptomatic and asymptomatic. Secondary aims were to evaluate how frequently SARS-CoV-2 infection is undiagnosed in adults from diverse countries and describe the relationship between COVID vaccination rate and SARS-CoV-2 seropositivity at a country level.

## MATERIALS AND METHODS

This seroepidemiologic analysis is a substudy of the Prospective Urban Rural Epidemiology (PURE) study. PURE is an international prospective cohort study in which community-dwelling adults aged 35–70 years from urban and rural communities were enrolled and thoroughly characterized (5). While the countries and communities involved were not intended to be globally or nationally representative, respectively, participants were recruited using strategies aimed to ensure they were representative of their communities. Specific sampling strategies varied according to the setting; however, these sampling approaches have produced a cohort with national mortality rates that are closely correlated with those recorded in national databases (5).

The present PURE substudy was conducted in alignment with the World Health Organization Seroepidemiology Technical Working Group’s recommendations on studies of SARS-CoV-2 (6). When the COVID-19 pandemic was declared on 11 March 2020, a questionnaire to be administered to PURE study participants was developed to solicit information on COVID-19 infection, the method of diagnosis, and COVID-19 vaccination. These data were collected from 47,350 participants in 22 countries (Fig. S1). Blood samples were also collected. This was performed in 10,749 participants from 11 countries where the investigators had the capacity to undertake blood collection within the constraints on research imposed by local governance in response to the pandemic. No specific sampling strategy was implemented to select participants for blood collection. Rather, sites approached individuals who were existing PURE study participants in a pragmatic manner. All participants provided written informed consent.

### Characterizing participants

PURE participants have been thoroughly characterized at enrolment and at three-year intervals subsequently. The following participant characteristics were collected at enrolment in the PURE study: age, sex, education, employment, tobacco and alcohol use, diet, self-reported wheezing in the prior six months, morning cough and chest tightness, and morbidities. Physical activity was measured using the International Physical Activity Questionnaire, which includes both work-related and recreational activity. Physical activity was categorized as low, medium, and high (7). The forced expiratory lung volume in 1 s (FEV1), forced vital capacity (FVC), and handgrip strength were measured as previously described (8).

Diabetes was identified by self-report, use of blood glucose-lowering medications, or a fasting glucose concentration ≥7 mmol/L (for which blood was collected in 79% of participants). Diet quality was assessed using the Alternative Healthy Eating Index, as previously reported (9). Higher scores are associated with a lower risk of several diseases. Cardiovascular disease was considered any self-reported coronary artery disease, myocardial infarction, stroke, or heart failure. Chronic lung disease was considered as self-reported chronic obstructive pulmonary disease or asthma.

After the baseline visit, participants have been followed every three years until present, when the blood specimens for the current analysis were collected. During follow-up, many baseline characteristics were re-measured. Therefore, pre-pandemic information on participants’ alcohol and tobacco use, employment, number of household members, morbidities (asthma, chronic obstructive pulmonary disease, cardiovascular disease, diabetes, hypertension, and cancer), physical activity, blood pressure, body mass index (BMI), waist circumference, FEV1, and handgrip strength was recently collected. For the purposes of this analysis, the participant’s most recent pre-pandemic characteristics were considered as the exposures of interest.

### Blood specimen processing and analysis for SARS-CoV-2 serology

Within 1 hour of blood collection, specimens were centrifuged at 1,500 × *g* for 15 minutes. The serum was aliquoted and stored at −70°C for subsequent batch analysis. Specimens collected in Canada (*n* = 954), Colombia (*n* = 714), the United Arab Emirates (*n* = 161), Chile (*n* = 253), Argentina (*n* = 505), Poland (*n* = 261), Malaysia (*n* = 137), and the Philippines (*n* = 437) were shipped on dry ice to a core laboratory at McMaster University, Hamilton, Canada. These samples were analyzed using the Biorad Platelia (#72710) assay to detect IgG, IgM, and IgA against viral nucleocapsid proteins. This assay has reported a sensitivity and specificity of 98% and 99%, respectively (10). Specimens collected from three sites in India (*n* = 4,693) were shipped on dry ice to a core laboratory in Bangalore, India. There, they were analyzed using the Abbott Architect assay for the detection of IgG against viral nucleocapsid protein, which has a sensitivity of 94% and a specificity of 100% (11). One participating site in Türkiye conducted serologic analyses on specimens collected at that site (*n* = 1,235) using the Roche Elecsys assay, which detects IgG antibodies against viral spike protein receptor binding domain with a sensitivity of 86% and a specificity of 100% (11, 12).

### Statistical analysis

Each participant’s sex and education levels were assumed to be the same at the time of blood collection for this substudy as at their enrolment in the overall PURE cohort. Alcohol and tobacco use, employment, number of household members, morbidities (asthma, chronic obstructive pulmonary disease, cardiovascular disease, diabetes, hypertension, cancer), physical activity levels, blood pressure, body mass index, waist circumference, spirometry, and handgrip strength were treated as time-varying co-variates; for this analysis, the most recent pre-pandemic value of each of these co-variates was used. For analysis, the Alternative Health Eating Index was divided by quartile. The characteristics of those with and without serologic evidence of SARS-CoV-2 infection were compared using the analysis of variance for continuous variables and the $\chi^{2}$ test for categorical variables.

Characteristics that were independently associated with seropositivity were identified by multivariable logistic regression. Characteristics that were associated with seropositivity at a *P*-value threshold of <0.2 were included in the multivariable model (13), which was performed by simultaneous forced entry (14).

COVID vaccination rates and SARS-CoV-2 seropositivity rates were stratified by country and adjusted by logistic regression for the length of time from the start of the pandemic (11 March 2020) until the date of blood collection for each individual. The relationship between national vaccination and seropositivity rates was evaluated by linear regression.

## RESULTS

### Individual characteristics associated with SARS-CoV-2 seropositivity

Serum was collected from 10,921 participants in 11 countries at a median of 19 months (25th–75th percentile, 13–22 months) after the pandemic declaration (corresponding to October 2021, April 2021–January 2022). Of these individuals, 2,202 who had been vaccinated against COVID-19 prior to blood collection using an inactivated viral vaccine (Sinopharm, Sinovac, or Covaxin), or who had received any COVID-19 vaccination prior to serologic analysis using the Roche Elecsys assay were excluded because, in these circumstances, prior SARS-CoV-2 infection cannot be distinguished from the serologic effects of vaccination.

The characteristics of the remaining 8,719 participants stratified by seropositivity (*n* = 3,009; 35%) versus seronegativity (*n* = 5,710; 65%) to SARS-CoV-2 are presented in Table 1. Seropositive individuals were younger, more often female, less educated, had >2 cohabitants in their homes more frequently, were less likely to drink alcohol, and had worse diet quality. Chronic lung disease and a history of cancer were more common among seronegative participants. FEV1 and FVC were lower, but the FEV1/FVC ratio was higher among seropositive individuals.

**TABLE 1:** Participant characteristics stratified by seropositivity versus seronegativity for SARS-CoV-2^*a*^

Characteristic	Seropositive *N* = 3,009	Seronegative *N* = 5,710	*P*-value
Country income level			<0.0001
HIC	212 (7)	1,081 (19)	
UMIC	683 (23)	917 (16)	
LMIC	690 (23)	514 (9)	
LIC	1,424 (47)	3,198 (56)	
Current age, years	61.8 ± 8.8	63.2 ± 9.0	<0.0001
Female	1,920 (64)	3,289 (58)	0.001
Number in household			<0.0001
Alone	155 (6)	373 (7)	
1–2 cohabitants	1,034 (40)	2,393 (44)	
>2 cohabitants	1,403 (54)	2,667 (49)	
Education			<0.0001
Primary	1,484 (50)	2,662 (47)	
Secondary	965 (32)	1,576 (28)	
>Secondary	550 (18)	1,439 (25)	
Employed	1,685 (57)	3,337 (59)	0.086
Alcohol			<0.0001
Never	2,044 (68)	3,709 (65)	
Former	155 (5)	207 (4)	
Current	808 (27)	1,789 (31)	
Tobacco			0.12
Never	2,075 (69)	3,807 (67)	
Former	365 (12)	746 (13)	
Current	566 (19)	1,142 (20)	
AHEI			<0.001
Quartile 1	889 (31)	1,296 (24)	
Quartile 2	754 (26)	1,498 (28)	
Quartile 3	743 (25)	1,487 (27)	
Quartile 4	516 (18)	1,170 (21)	
Baseline wheeze	241 (8)	442 (8)	0.66
Morning cough	216 (7)	374 (7)	0.26
Diabetes	241 (8)	371 (7)	0.009
Hypertension	511 (17)	858 (15)	0.017
CVD	154 (5)	317 (6)	0.239
COPD	43 (2)	124 (3)	0.007
Asthma	127 (4)	315 (6)	0.008
Cancer	87 (3)	264 (5)	<0.001
ACE-I	124 (4)	217 (4)	0.46
Physical activity			0.57
Low	333 (12)	660 (12)	
Medium	892 (31)	1,643 (31)	
High	1,628 (57)	2,988 (57)	
Body mass index, kg/m^2^			
Mean ± SD	25.1 ± 7.9	25.0 ± 8.7	0.36
≥25	1,403 (49)	2,476 (49)	0.015
FEV1, L	2.12 ± 0.75	2.21 ± 0.77	<0.0001
FVC, L	2.60 ± 0.92	2.77 ± 0.93	<0.0001
FEV1/FVC ratio	0.87 ± 0.10	0.86 ± 0.10	0.003
Grip strength, kg	26.1 ± 11.6	25.6 ± 12.8	0.057
Received COVID vaccine	1,241 (44)	2,389 (42)	0.10

^*a*^ Statistics are mean ± standard deviation (SD) or count (column percentage). Cardiovascular disease (CVD) included coronary artery disease, myocardial infarction, stroke, or heart failure. Chronic lung disease included chronic obstructive pulmonary disease and asthma. ACE-I = angiotensin converting enzyme-inhibitor; AHEI = Alternative Healthy Eating Index; CVD = cardiovascular disease; FEV1 = forced expiratory volume in 1 s; FVC = forced vital capacity; HIC = high-income countries; LIC = low-income countries; LMIC = low-middle income countries; UMIC = upper middle-income countries.

In the multivariable model (Table 2), characteristics that were independently associated with SARS-CoV-2 seropositivity were younger age (odds ratio, OR; 95% confidence interval, CI, per five-year increase in age: 0.95; 0.91–0.98, *P* = 0.006) and BMI > 25 kg/m^2^ (OR; 95% CI: 1.16; 1.01–1.34, *P* = 0.037). Current tobacco use (OR; 95% CI: 0.83; 0.70–0.97, *P* = 0.021) and COVID-19 vaccination (OR; 95% CI: 0.70; 0.60–0.82, *P* < 0.001) were associated with a 30% lower risk of seropositivity.

**TABLE 2:** Univariable and multivariable logistic regression models for SARS-CoV-2 seropositivity^*a*^

Characteristic	Unadjusted		Adjusted for country		Multivariable model	
	OR (95% CI)	*P*-value	OR (95% CI)	*P*-value	Adjusted OR (95% CI)	*P*-value
Age, per 5-year increase	0.91 (0.89–0.93)	<0.001	0.94 (0.92–0.97)	<0.001	0.95 (0.91–0.98)	0.006
Male	0.77 (0.70–0.84)	<0.001	0.90 (0.82–0.99)	0.027	0.90 (0.76–1.06)	0.21
Number in household						
Alone	Ref.		Ref.		Ref.	
1–2 cohabitants	1.04 (0.85–1.27)	0.70	1.11 (0.90–1.37)	0.33	1.08 (0.84–1.38)	0.57
>2 cohabitants	1.27 (1.04–1.54)	0.020	1.27 (1.02–1.57)	0.030	1.24 (0.96–1.61)	0.10
Education						
Primary	Ref.		Ref.		Ref.	
Secondary	1.10 (0.99–1.22)	0.072	1.11 (1.00–1.24)	0.072	1.12 (0.97–1.30)	0.12
>Secondary	0.69 (0.61–0.77)	<0.001	0.92 (0.79–1.06)	0.24	0.90 (0.73–1.10)	0.30
Employed	0.92 (0.85–1.01)	0.087	1.06 (0.96–1.17)	0.22	−	−
Alcohol use						
Never	Ref.		Ref.		Ref.	
Former	1.36 (1.10–1.68)	0.10	1.19 (0.94–1.51)	0.15	1.35 (0.99–1.83)	0.061
Current	0.82 (0.74–0.90)	<0.001	0.91 (0.80–1.04)	0.18	1.02 (0.86–1.22)	0.83
Tobacco use						
Never	Ref.		Ref.		Ref.	
Former	0.90 (0.78–1.03)	0.12	0.95 (0.81–1.11)	0.52	0.98 (0.81–1.20)	0.87
Current	0.91 (0.81–1.02)	0.10	0.85 (0.76–0.96)	0.009	0.83 (0.70–0.97)	0.021
AHEI, per quartile increase	0.87 (0.84–0.91)	<0.001	0.96 (0.91–1.00)	0.069	0.96 (0.90–1.02)	0.16
History of wheeze at baseline	1.04 (0.88–1.22)	0.66	0.97 (0.82–1.15)	0.74	−	−
History of morning cough at baseline	1.10 (0.93–1.31)	0.26	0.76 (0.63–0.91)	0.004	0.80 (0.62–1.04)	0.095
Hypertension	1.16 (1.03–1.30)	0.017	0.94 (0.82–1.07)	0.33	−	−
Diabetes	1.25 (1.06–1.48)	0.009	1.11 (0.92–1.33)	0.27	−	−
Physical activity					−	−
Low	Ref.		Ref.			
Medium	0.90 (0.79–1.03)	0.12	0.96 (0.83–1.11)	0.60		
High	0.75 (0.67–0.84)	<0.001	0.93 (0.80–1.08)	0.35		
BMI > 25 kg/m^2^	1.12 (1.02–1.22)	0.015	1.10 (0.99–1.23)	0.069	1.16 (1.01–1.34)	0.037
Cardiovascular disease	0.92 (0.75–1.12)	0.39	0.98 (0.79–1.22)	0.86	−	−
COPD	0.62 (0.44–0.88)	0.008	0.88 (0.60–1.29)	0.51	−	−
Asthma	0.75 (0.61–0.93)	0.009	0.82 (0.66–1.03)	0.083	0.85 (0.65–1.12)	0.25
History of cancer	0.61 (0.48–0.79)	<0.001	0.97 (0.74–1.27)	0.81	−	−
ACE-I use	1.09 (0.87–1.36)	0.46	0.98 (0.76–1.26)	0.87	−	−
FEV1, per 1L increase	0.87 (0.82–0.92)	<0.001	0.97 (0.90–1.04)	0.37	−	−
FVC, per 1L increase	0.82 (0.78–0.87)	<0.001	0.91 (0.85–0.97)	0.006	0.96 (0.87–1.05)	0.36
Handgrip strength, per 5 kg increase	0.95 (0.93–0.97)	<0.001	1.01 (0.98–1.04)	0.53	−	−
COVID-19 vaccination	1.08 (0.98–1.18)	0.10	1.19 (1.07–1.33)	0.002	0.70 (0.60–0.82)	<0.001
Months from pandemic onset until blood collection	1.11 (1.10–1.12)	<0.001	1.14 (1.13–1.15)	<0.001	1.15 (1.14–1.17)	<0.001

^*a*^ AHEI = Alternative Healthy Eating Index (higher values are associated with lower risk of some diseases); BMI = body mass index; COPD = chronic obstructive pulmonary disease; FEV1 = forced expiratory volume in 1 s; FVC = forced vital capacity; Ref. = reference category. The multivariable model is adjusted for age, sex, education levels, number of co-habitants, alcohol and tobacco use, diet quality (AHEI), history of cough, BMI, FVC, COVID vaccination, time from the pandemic declaration until blood collection, and country.

Sensitivity analyses were performed by repeating the multivariable model stratified by the type of SARS-CoV-2 assay used. The findings were generally similar to the overall model (not presented).

Chewing tobacco has been associated with an increased risk of SARS-CoV-2 infection among women from Bangladesh (15). Data on chewing tobacco were collected from a subset of 4,242 participants from India, 347 (8%) of whom reported current or prior chewing tobacco. There was no association between chewing tobacco and SARS-CoV-2 seropositivity (unadjusted OR, 95% CI: 1.09, 0.86–1.38; *P* = 0.49).

### Awareness of SARS-CoV-2 infection

**Fig 1:**
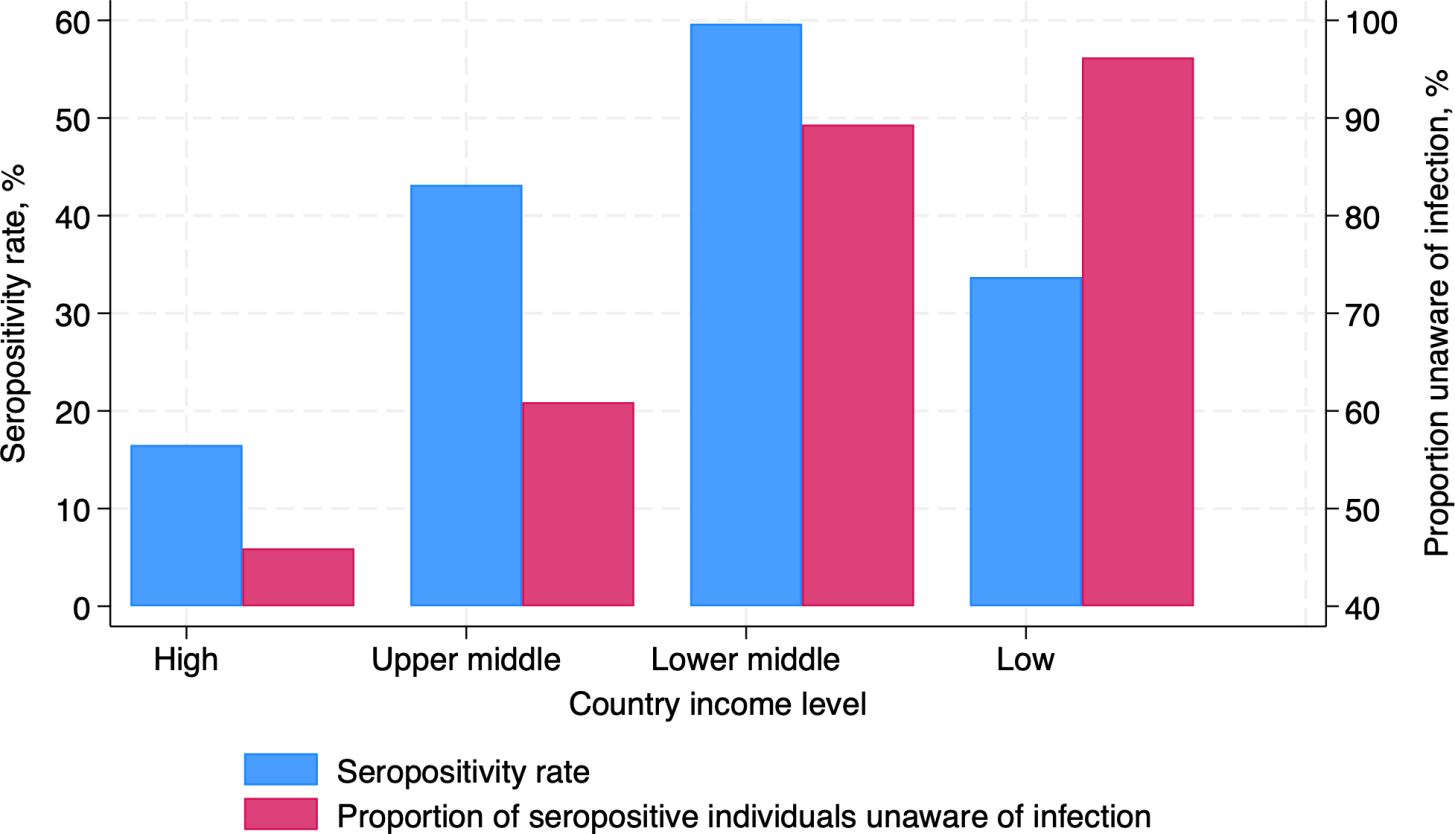
Bar graphs representing the proportion of 8,764 participants who were seropositive for SARS-CoV-2 (in blue) between November 2019 and September 2022, and the proportion of seropositive participants who were aware of SARS-CoV-2 infection (in red) stratified by country income level. Estimates were adjusted for the time from pandemic declaration to blood collection.

At the time of blood collection, 2,524 (83%) seropositive participants reported not having had COVID-19 and were unaware of having been infected with SARS-CoV-2. Analyses were performed to characterize those who were aware of having been infected (*n* = 502) versus those who were seropositive but unaware of infection (*n* = 2,524). Differences in these two groups are presented in Table 3. Disparities in infection awareness were observed across different country income levels. After adjustment for country income level and time from pandemic declaration to the date of blood collection, 54% of seropositive individuals in high-income countries were aware of prior infections. This proportion fell to 39%, 11%, and 3% in upper-middle, lower-middle, and low-income countries, respectively (Fig. 1). Those living with one or two other people were more likely to be aware of infection, while those living with >2 others were less likely to be aware. Education was positively associated with awareness of infection. Current alcohol drinkers, ex-smokers, those with healthier diets, a history of hypertension, angiotensin converting enzyme-inhibitor (ACE-I) use or cancer, elevated BMI, higher FEV1 or FVC, or COVID-19 vaccination were more likely to be aware of infection. Those with an early morning cough in the last six months or with high physical activity levels were less likely to be aware of infection.

**TABLE 3:** Seropositive participants stratified by awareness of infection versus unawareness^*a*^

Characteristic	Unaware of infection *N* = 2,524	Aware of infection *N* = 502	*P*-value
Country income level			<0.001
HIC	98 (4)	114 (23)	
UMIC	416 (17)	267 (53)	
LMIC	614 (24)	76 (15)	
LIC	1379 (55)	54 (9)	
Current age, years	61.9 ± 8.9	61.5 ± 8.7	0.41
Female	1,588 (63)	332 (66)	0.24
Number in household			<0.001
Alone	124 (6)	31 (6)	
1–2 cohabitants	798 (38)	236 (49)	
>2 cohabitants	1,184 (56)	219 (45)	
Education			<0.001
Primary	1,279 (51)	2,105 (41)	
Secondary	808 (32)	157 (31)	
>Secondary	410 (17)	140 (28)	
Employed	1,418 (57)	267 (55)	0.32
Alcohol			<0.001
Never	1,783 (71)	261 (52)	
Former	128 (5)	27 (5)	
Current	594 (24)	214 (43)	
Tobacco			<0.001
Never	1,749 (70)	326 (65)	
Former	262 (10)	103 (21)	
Current	494 (20)	72 (14)	
AHEI			<0.001
Quartile 1	739 (31)	150 (31)	
Quartile 2	634 (26)	120 (25)	
Quartile 3	645 (27)	98 (20)	
Quartile 4	395 (16)	121 (25)	
Baseline wheeze	202 (8)	39 (8)	0.82
Morning cough	188 (8)	28 (6)	0.12
Diabetes	195 (8)	46 (9)	0.30
Hypertension	408 (16)	103 (21)	0.021
CVD	127 (5)	27 (5)	0.75
COPD	33 (2)	10 (2)	0.92
Asthma	102 (4)	25 (5)	0.36
Cancer	56 (2)	31 (6)	<0.001
ACE-I	90 (4)	34 (7)	0.001
Physical activity			0.015
Low	271 (12)	62 (13)	
Medium	723 (30)	169 (36)	
High	1,387 (58)	241 (51)	
Body mass index, kg/m^2^			
Mean ± SD	24.5 ± 8.2	28.3 ± 5.3	<0.001
≥25	1,048 (44)	355 (72)	<0.001
FEV1, L	2.05 ± 0.72	2.50 ± 0.80	<0.001
FVC, L	2.51 ± 0.88	3.02 ± 0.96	<0.001
FEV1/FVC ratio	0.87 ± 0.10	0.87 ± 0.11	0.88
Grip strength, kg	26.6 ± 10.6	24.1 ± 15.4	<0.001
Received COVID vaccine	872 (38)	369 (74)	<0.001

^*a*^ Statistics are mean ± standard deviation (SD) or count (column percentage). Cardiovascular disease (CVD) included coronary artery disease, myocardial infarction, stroke, or heart failure. Chronic lung disease included chronic obstructive pulmonary disease and asthma. ACE-I = angiotensin converting enzyme-inhibitor; AHEI = Alternative Healthy Eating Index (higher values are associated with lower risk of some diseases); CVD = cardiovascular disease; FEV1 = forced expiratory volume in 1 s; FVC = forced vital capacity; HIC = high-income countries; LIC = low-income countries; LMIC = lower middle-income countries; UMIC = upper middle-income countries.

A multivariable logistic regression model was performed to identify factors associated with awareness of SARS-CoV-2 infection (Table S1). Among seropositive individuals, characteristics that were independently associated with infection awareness were >2 co-habitants (OR; 95% CI: 1.95; 1.17–3.25); higher FEV1 (OR per 1L increase; 95% CI: 1.42; 1.06–1.91) and COVID vaccination (OR; 95% CI: 1.42; 1.05–1.91). Current alcohol use was associated with a decreased likelihood of being aware of infection (OR, 95% CI: 0.58, 0.38–0.88) as compared with never drinking. Country income level was strongly associated with awareness of the SARS-CoV-2 infection. As compared with low-income countries, seropositive individuals in lower middle-income countries (OR; 95% CI: 10.4, 5.5–19.0); upper middle-income countries (OR; 95% CI: 30.4, 16.9–54.4) and high-income countries (OR; 95% CI: 48.8, 25.1–94.8) were more likely to be aware of infection.

### Country-level seropositivity and vaccination rates

**Fig 2:**
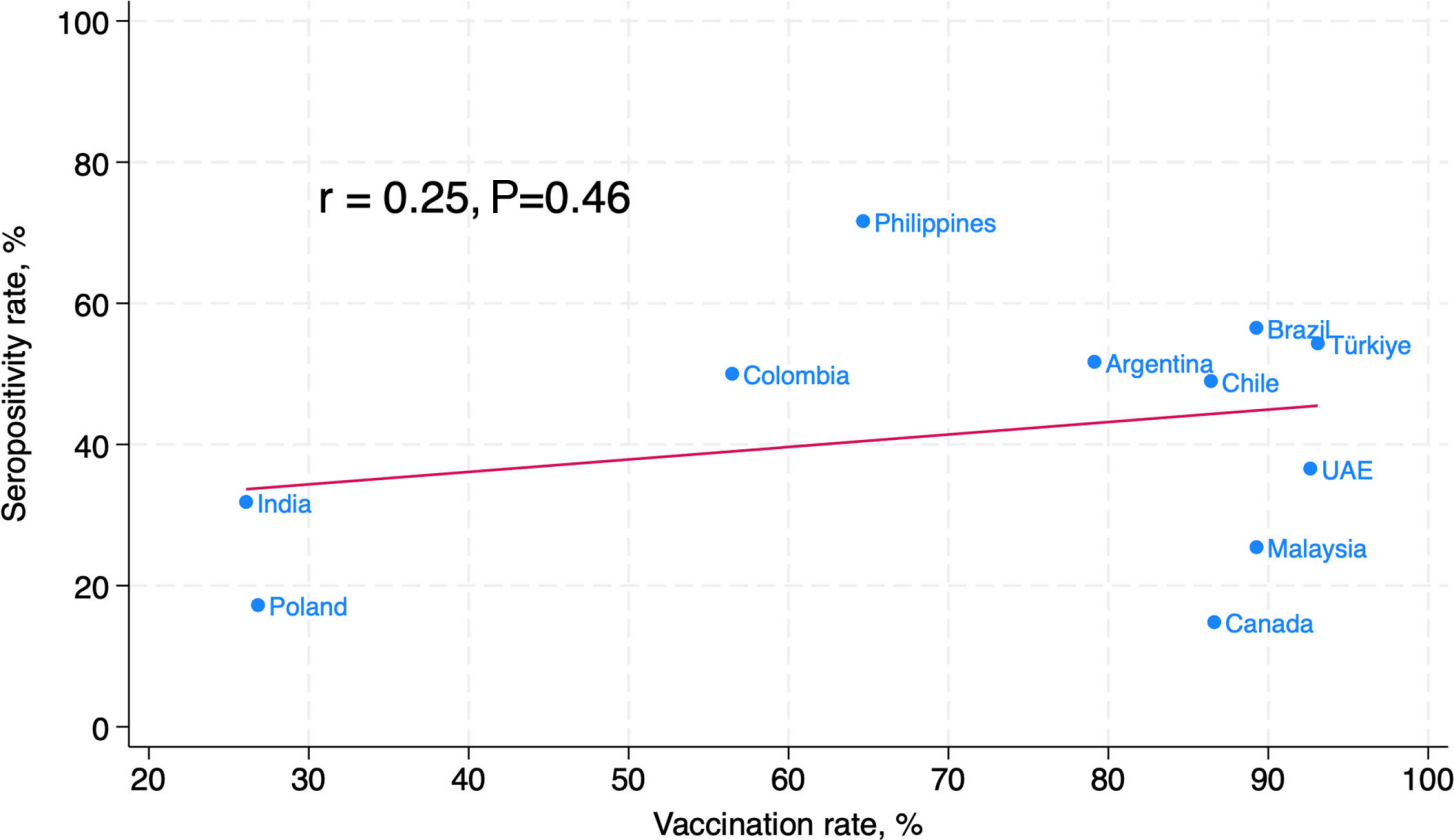
Scatterplot illustrating the relationship between country-level COVID-19 vaccination rates and SARS-CoV-2 seropositivity rates. Rates are adjusted for the time from pandemic declaration to the date of participants’ blood collection.

There were wide variations between countries in seropositivity (Fig. 1) and vaccination rates even after adjusting for time from the pandemic onset to the date of blood collection. At a population level, there was no evidence of a relationship between national COVID vaccination and seropositivity rates (r = 0.25, *P* = 0.46) (Fig. 2).

## DISCUSSION

The major findings from this international seroepidemiologic study, including participants from high-, middle-, and low-income countries, are (i) BMI >25 kg/m^2^ was associated with an increased risk of SARS-CoV-2 infection, while current smoking and COVID-19 vaccination were associated with a reduced risk of infection; (ii) 83% of those with evidence of SARS-CoV-2 infection were unaware of infection, with lower-income countries featuring higher seropositivity rates and more infection unawareness; and (iii) at a population level, COVID-19 vaccination was not associated with reduced rates of seropositivity.

### Risk factors for SARS-CoV-2 infection

There are limited data on individual characteristics that predispose to SARS-CoV-2 infection. In a rigorous study, 4,142 participants from 1,394 households in the United States underwent bi-weekly nasal swabs for six months to detect SARS-CoV-2 infection. Consistent with our observations, an elevated body mass index was a risk factor for infection, while asthma was not (16). Our findings extend this study by including a larger range of potential confounding factors and participants from diverse countries.

Two seroepidemiologic studies from the United Kingdom (one in healthcare workers from a single institution and the other from antenatal blood samples) found higher rates of SARS-CoV-2 infection among younger adults as compared with older adults (17, 18). We also found that, at univariable level, younger age was associated with a higher risk of infection. However, this association was attenuated after adjustment, suggesting that other individual characteristics may account for the relationship between age and SARS-CoV-2 infection. These two British seroepidemiologic studies also demonstrated that Asian or Black ethnicity was associated with a higher risk of SARS-CoV-2 infection. We did not evaluate whether ethnicity influences the risk of SARS-CoV-2 infection because ecological factors are likely to play a major role in the differences in seropositivity observed among participating countries.

We were unable to replicate the findings from a seroepidemiologic study of women at a single site in Bangladesh, which demonstrated that chewing tobacco was associated with an increased risk of seropositivity. In contrast, we found that any current tobacco use was associated with a decreased risk of SARS-CoV-2. This observation is consistent with the results of a systematic review examining the relationship between smoking and SARS-CoV-2 (19). Our research extends on these previous data because we avoid several potential sources of bias. Case ascertainment in our study was objective and did not depend on the individual seeking a diagnostic test for COVID-19, which may have led to ascertainment biases in other papers. Because our data on tobacco use were collected in a prospective manner prior to the pandemic, they are less susceptible to recall or responder biases. The inverse association between smoking and SARS-CoV-2 seropositivity appears counter-intuitive because smoking impairs the immune system and increases the risk of several types of pneumonia (20). However, several other serologic studies have found that SARS-CoV-2 infection is less in smokers (21–25). Smoking is known to cause inflammation in airways. Whether this chronic inflammation can protect against SARS-CoV-2 infection is unknown. However, we cannot exclude unmeasured confounding accounting for our observation, and more specific research is needed on the relationship between smoking and SARS-CoV-2 infection.

Research from the Nurses’ Health Study II and the Health Professionals Follow-up Study suggested that a healthier diet might be associated with a lower risk of COVID-19 (26). Case ascertainment bias cannot be excluded in this paper because testing for SARS-CoV-2 infection was not performed systematically. At the univariable level, we also found that a healthier diet was associated with a lower risk of SARS-CoV-2 seropositivity. However, this association was no longer present after multivariable adjustment, suggesting that other characteristics might explain the inverse relationship between diet quality and SARS-CoV-2 infection risk.

Our finding of an association between overweight/obesity (BMI > 25 kg/m^2^) and SARS-CoV-2 infection is potentially important. Obesity is known to be a risk factor for poor outcomes in those with symptomatic COVID-19. We have proposed that COVID-19 vaccination prioritization based on the presence of obesity would be an efficient way of allocating vaccines in conditions of scarcity (27). Our current research supports this idea.

Obesity negatively impacts immune function. There is an inverse association between body mass index and neutralizing antibody response to COVID-19 vaccination (28). This observation has two implications: (i) our estimate of the risk of SARS-CoV-2 infection in the overweight/obese may be an underestimate if their serologic response to infection is less than those with a BMI 20–25 kg/m^2^; and (ii) if obesity leads to reduced mucosal immune responses to SARS-CoV-2, it may represent a mechanism by which elevated BMI leads to an increased risk of infection in those exposed to the virus.

### Unrecognized SARS-CoV-2 infection

Systematic reviews of studies that reported screening for SARS-CoV-2 concluded that at least one in every three to five infections is asymptomatic (29, 30). However, the studies included in these systematic reviews were limited: some were reports of screening performed on ships, and others were screening programs performed in single institutions. There were few population-based studies where SARS-CoV-2 infection was ascertained systematically and no data from multiple countries.

In our study, we found that 83% of those with evidence of SARS-CoV-2 infection were unaware of the infection. While this proportion is not synonymous with the rate of asymptomatic infection (because individuals may have been symptomatic with COVID-19 but never received a diagnostic test), it does indicate that the large majority of those infected remain undiagnosed. We found a higher rate of unrecognized SARS-CoV-2 infection than a seroepidemiologic study from Athens, Greece, in which 49% of serologically confirmed cases had no history of COVID-19 infection (31). This is important because those with an undiagnosed infection could be the main source from which SARS-CoV-2 spreads throughout populations. Our finding leads to the hypothesis that greater availability of SARS-CoV-2 rapid antigen test kits might increase the recognition of infection, including asymptomatic infection, and so help control viral dissemination. This hypothesis is unproven, however.

We observed an inverse relationship between country income level and seropositivity rates, and a positive association between country income level and awareness of infection among those who were seropositive. This finding is consistent with health systems that imposed more restrictive measures to limit SARS-CoV-2 spread and that made viral testing more widely available and accessible in higher income countries. A systematic review of seroprevalence studies published between 1 January 2020 and 20 May 2022 identified several sources of data to inform rates of SARS-CoV-2 seropositivity, including blood donors, residual sera, household and community samples, pregnant women, persons from slums, and the general population (32). These data indicated unsurprisingly that global seroprevalence has increased over time. Our study demonstrated the same at an individual rather than a population prevalence level. Thus, our findings have face validity. The systematic review also showed that as of September 2021, the global seroprevalence was 59.2% (95% CI: 56.1%–62.2%). In contrast, we found that at a median date of October 2021, the seropositivity rate was 30%. This difference might be explained by studies in the systematic review that included individuals whose serology was performed after vaccination. COVID-19 vaccines that use whole SARS-CoV-2 as the immunogen will elicit antibody responses to spike protein, which may lead to false positives. In our analysis, we excluded those vaccinated with inactivated viral vaccines because the serologic effects of vaccination cannot be distinguished from SARS-CoV-2 infection.

### COVID-19 vaccination and SARS-CoV-2 infection risk

COVID-19 vaccines reduce the risk of severe disease. The evidence that they reduce SARS-CoV-2 infection (as distinct to reducing severe disease after infection has occurred) is more limited, especially in a population setting. We observed an interesting paradox: while vaccination was strongly associated with a reduced risk of SARS-CoV-2 infection, at a country level, we did not find an association between vaccination rates and infection rates. However, with only 10 participating countries, our study may have lacked the power to demonstrate the population-level effects of COVID-19 vaccination in reducing SARS-CoV-2 infection rates. An important implication of our finding is that COVID-19 vaccination is likely to be an important way for individuals to reduce their risk of disease, but further research is needed to understand the optimal ways to minimize or slow population infection rates.

### Strengths and limitations

The strengths of our study are that it is a large, multi-national study with representation in high-, middle-, and low-income countries. Ascertainment of SARS-CoV-2 infection was performed serologically, which avoids the ascertainment bias that occurs in studies where COVID-19 occurrence is measured in individuals seeking COVID-19 testing. Ascertainment bias may be common in studies without systematic serologic testing because one in three SARS-CoV-2 cases (more in our data) is asymptomatic (30, 33). We collected a broader range of exposures than in any previous COVID-19 study, and these exposures were recorded prior to the pandemic declaration. This avoids reverse causation, whereby SARS-CoV-2 can alter individuals’ phenotypes rather than their phenotypes predisposing to SARS-CoV-2 infection.

The major limitation of our study is inherent in the use of serology to ascertain SARS-CoV-2 infection, as antibody levels decrease over time and may fall below the limit of detection at the time of blood collection in those previously infected, resulting in falsely negative tests (34), especially as the performance of serologic tests in those whose infection was remote is less certain (35). In addition, owing to reasons of availability and import limitations, we were unable to use the same assays in all countries. Also, our sampling frame may not be representative of all populations, including children, young adults, and residents from long-term care. In addition, while the overall PURE study sample is intended to represent the communities in which participants reside, the pragmatic way in which blood was collected in this substudy could have resulted in a non-representative study cohort. Data on access to as well as negative COVID-19 testing were not collected, so for those with unrecognized SARS-CoV-2 infection, the reasons (negative test or no access to testing) are not clear. Data on viral symptoms were not collected in individuals who did not have a positive COVID-19 test, so we cannot distinguish asymptomatic from symptomatic infection. Finally, we cannot exclude the possibility of unmeasured confounding and chance associations. However, our data are the most robust available to date owing to the sample size and range of exposures evaluated.

### Conclusions

Risk factors for SARS-CoV-2 infection include younger age, overweight/obesity (BMI > 25 kg/m^2^), and living with >2 other people, while current smoking is associated with lower infection risk. COVID-19 vaccination is associated with a decreased risk of recognized or unrecognized SARS-CoV-2 infection for individuals. Country income level is strongly associated with recognition of infection.

## Data Availability

Individual-level data will not be made publicly available because further analyses are planned using the data and because consent has not been obtained from study participants to release these data to researchers who are not study investigators. On reasonable request, aggregate data will be made available. Study case report forms, the laboratory procedures manual, and statistical code will also be shared on request.

## SUPPLEMENTAL MATERIAL

Supplemental file spectrum.01492-23-s0001.docx Supplemental material.

ASM does not own the copyrights to Supplemental Material that may be linked to, or accessed through, an article. The authors have granted ASM a non-exclusive, world-wide license to publish the Supplemental Material files. Please contact the corresponding author directly for reuse.
